# The Technical Challenges for Applying Unsaturated Soil Sensors to Conduct Laboratory-Scale Seepage Experiments

**DOI:** 10.3390/s22103724

**Published:** 2022-05-13

**Authors:** Guanxi Yan, Thierry Bore, Habibullah Bhuyan, Stefan Schlaeger, Alexander Scheuermann

**Affiliations:** 1 School of Civil Engineering, University of Queensland, St. Lucia, Brisbane, QLD 4072, Australia; t.bore@uq.edu.au (T.B.); h.bhuyan@uq.edu.au (H.B.); a.scheuermann@uq.edu.au (A.S.); 2 Science-Engineering-Measurement, Sceme.de GmbH, HRB 7181 Amtsgericht Lemgo, 32805 Horn-Bad Meinberg, Germany; stefan.schlaeger@sceme.de

**Keywords:** unsaturated soil suction, soil moisture content, sensor technique, tensiometer, spatial TDR, data-logging system, signal processing

## Abstract

Although many unsaturated soil experiments have successfully delivered positive outcomes, most studies just concisely illustrated sensor techniques, because their main objectives focused on bridging research gaps. Inexperienced research fellows might rarely follow up those techniques, so they could encounter very trivial and skill-demanding difficulties, undermining the quality of experimental outcomes. With a motivation to avoid those, this work introduces technical challenges in applying three sensor techniques: high precision tensiometer, spatial time-domain reflectometry (spatial TDR) and digital bench scales, which were utilized to measure three fundamental variables: soil suction, moisture content and accumulative outflow. The technical challenges are comprehensively elaborated from five aspects: the functional mechanism, assembling/manufacturing approaches, installation procedure, simultaneous data-logging configurations and post data/signal processing. The conclusions drawn in this work provide sufficient technical details of three sensors in terms of the aforementioned five aspects. This work aims to facilitate any new research fellows who carry out laboratory-scale soil column tests using the three sensors mentioned above. It is also expected that this work will salvage any experimenters having troubleshooting issues with those sensors and help researchers bypass those issues to focus more on their primary research interests.

## 1. Introduction

Since extending soil mechanics from saturated to unsaturated, the conventional seepage knowledge has been expanded from single phase seepage for groundwater to multiphase flow seepage in the vadose zone [1,2,3,4]. With this theoretical development, there has been a high demand for innovative sensor techniques and soil testing methods. Those sensor techniques were designed for measuring soil suction [5,6,7,8,9,10], soil moisture content [11,12,13,14,15,16,17], soil density/porosity [17,18,19,20,21,22], soil temperature [23,24,25], soil electrical conductivity [24,26,27], soil permeability/relative permeability [28,29,30,31,32], soil water retention curves [33,34,35,36,37,38], soil diffusivity [39,40], etc. With a blossoming development of those sensor techniques, the unsaturated soil testing methods have been simultaneously expanded from laboratory standard tests [32,38,41,42,43,44] to full-scale column tests with various boundary conditions [30,45,46,47,48,49,50,51,52] and field tests [13,18,53].

Many unsaturated soil experiments have been successfully conducted at the laboratory scale with many outstanding contributions in terms of equilibrium soil water retention behaviour under static/steady-state flow conditions and dynamic nonequilibrium soil water retention behaviour under transient flow conditions [4]. Nevertheless, before establishing and conducting those experiments, all selected sensors must be carefully calibrated, installed, and well developed with compatible data-logging systems [16,18,54,55,56,57,58]. Unfortunately, most soil experimental studies briefly presented sensor techniques and utilization without technical details, because their primary aims are bridging geotechnical research gaps rather than sensor techniques [4]. Hence, inexperienced researchers could face trivial technical difficulties that have never been reported and explained in detail. Lacking those technical details could jeopardize the reliability of experimental results and even waste time, labour, and costs. 

With an aim to rescue any fit into those cases and guide new experimenters to overcome those difficulties, based on previous experiences of applying full-scale soil column experiments in the geomechanics laboratory of the University of Queensland (UQ) [16,48,49,59,60,61], this work introduces three sensor techniques: high precision tensiometer, spatial time-domain reflectometry (Spatial TDR) and digital bench scales, which were used to measure three fundamental variables: soil suction, moisture content and accumulative outflow, with a user experience in terms of technical challenges encountered in applications from the following five perspectives: sensor working mechanism, assembling and manufacturing methods, installation approaches, corresponding data-logging platform development and signal/data post-processing.

The structure of this contribution will be distinctive from a standard one in the sequence of introduction, methodology, result, discussion and conclusion. Instead, despite the ongoing introduction, the second section briefing testing conditions and a technical summary at the end, this work consists of three main sections corresponding to three sensor techniques mentioned previously, each of which logically elaborates technic details as aforementioned five perspectives within several subsections. Finally, the conclusions are drawn to provide sufficient technical information on three sensors in terms of the five aspects mentioned above. 

This work aims to facilitate new research fellows who conduct laboratory-scale soil column tests using the three sensors mentioned above. It is also expected that this work will salvage any experimenters having troubleshooting issues with those sensors and help researchers bypass those issues to focus more on their primary research interests.

## 2. Sensors Applying Conditions

In order to elaborate on details for three sensor techniques, the applying condition in the form of full-scale soil column tests is briefly illustrated here. Eight full-scale soil column tests of 150–240 m long and 15 cm diameter have been carried out for six different sandy soils in the geomechanics laboratory of UQ. Furthermore, to avoid academic misconduct, one specified medium sand, which has never been and will never be shown in any other work on different research objectives, is presented here to demonstrate the sensor testing and applying conditions.

### 2.1. Material Selection and Specimen Installation

The only material selected for this demonstration is medium sand (sample code = 30/60), commercially accessed from Sebilco, Australia. According to technical specifications from the supplier, it should be river-washed sand with a mean diameter (*d_50_*) of 0.42 mm. For rigorousness, the fundamental properties were retested based on geotechnical testing standards of the grain size distribution (GSD) [62], specific unit weight (*G_s_*) [63] and maximum/minimum dry densities (*ρ_dry,max_*/*ρ_dry,min_*) [64]. 

**Figure 1:**
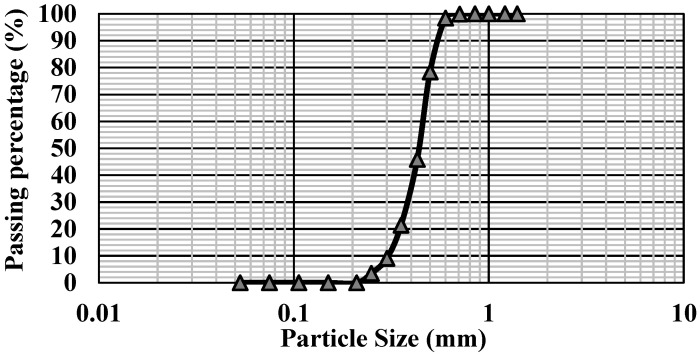
Grain size distribution (GSD) of Sebilco 30/60 sand.

The GSD of 30/60 sand and its specification are separately presented in Figure 1 and Table 1. According to Table 1, this sample confirmed *d_50_* = 0.42 mm with the *G_s_* = 2.65, indicating the most usual silica sand. Additionally, the coefficient of uniformity (*C_u_*) is 1.5, and the coefficient of curvature (*C_c_*) equals 1. Therefore, based on the unified soil classification system (USCS), this sample can be identified as a poor-graded uniform sand (SP). This sample was filled into a soil column of 1.5 m, introduced later, in a dry density (*ρ_dry_*) of 1.75 g/cm^3^, corresponding to *n* = 34% with *G_s_* = 2.65.

**Figure 2:**
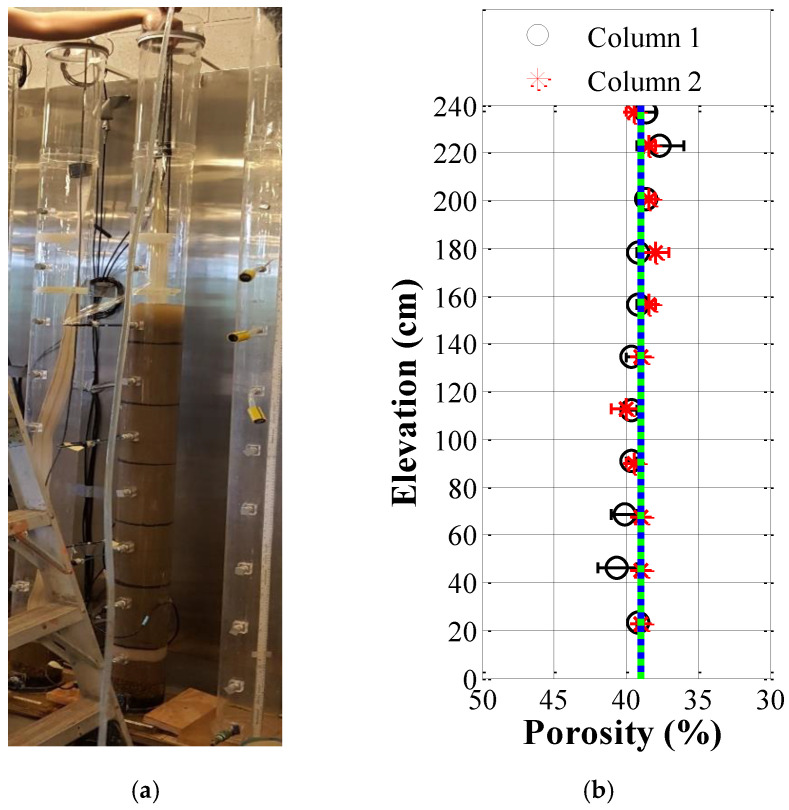
Homogeneity verification of sand columns: (**a**) the photo demo; (**b**) the porosity variations.

Yan, Li, Bore, Scheuermann, Galindo-Torres and Li [48] previously carried out density variation tests along 2.4 m soil columns to verify the homogeneity of such a specimen preparation. Figure 2a shows that each soil column was filled in every 20 cm, and then a thin layer of particles dyed in black was filled to mark the thickness of each layer. Once all samples loaded in, the settlement for each layer was rechecked and then used to determine corresponding dry densities. This specimen preparation could achieve two homogeneous sand profiles in *ρ_dry_* of 1.61–1.62 g/cm^3^ with standard deviations of ±0.03–0.05 g/cm^3^, corresponding to a mean *n* with a standard deviation of ±1%, as depicted in Figure 2b.

### 2.2. Soil Column Experiments

**Figure 3:**
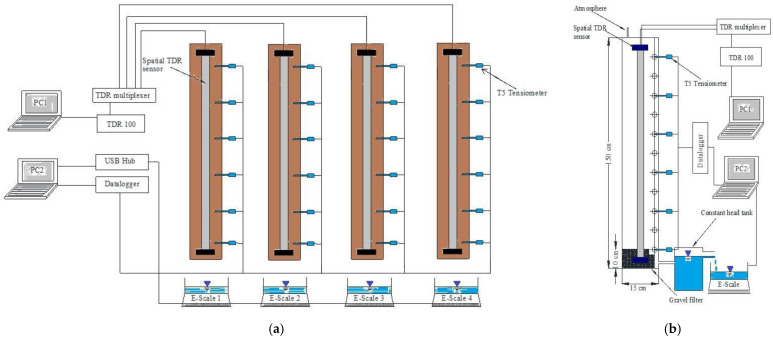
The schematic description of four Spatial TDR sensors—the flat ribbon cable (FRC), six high precision tensiometers (T5) and accumulative outflow logging scales and boundary conditions for the one-step in/outflow tests: (**a**) the overview of three data logging systems for four soil column tests; (**b**) the illustration of three data-logging systems for single soil column test of 150 cm in details.

These soil column in/outflow seepage experiments were conducted multiple times by authors in many prior studies for various research objectives in unsaturated soil water retention and seepage behaviour [16,48,49,59,60,61]. However, those research objectives are beyond the scope of the current work, so the main focuses are on sensor technologies. Figure 3a shows four spatial time-domain reflectometry (Spatial TDR) sensors—the flat ribbon cable (FRC) (technically supported by the Soil Moisture Group (SMG) at the University of Karlsruhe, Karlsruhe, Germany), six high precision T5 tensiometers (provided by UMS [65], Munich, Germany), and four digital bench scales (Ohaus Ranger 3000 R31P30 provided by Ohaus Ranger warehouse, Australia)—all integrated together to test the soil water retention and gas-water seepage in unsaturated soil simultaneously. In addition, all data in terms of signals could be sent and received from the dataloggers and TDR devices, later transferred to the digital computers for data storage and analysis. To be more specific, for each soil column test, Figure 3b illustrates the sensor locations (every 20 cm for tensiometer insertion and FRC prelocated in the centre), dimensions of polyvinyl chloride or acrylic columns in 150 cm length and 15 cm diameter, data-logging configurations for three sensors and an example of hydraulic boundary conditions applied to the column bottom. All tests were carried out at a constant ambient room temperature of 26 ± 2 °C, ensuring the soil water temperature was measured at 25 ± 1 °C. As the relative humidity could not be well controlled at a constant value in the laboratory, all open spaces on columns and water tanks were tightly covered by cling wraps with a few pinholes to avoid moisture and vapour exchanges between ambient air and soil/water inside (e.g., evaporation and condensation) while maintaining atmospheric pressure applied. Last, as for the configuration of sensors and scales regarding measurement and boundary conditions, details will be sufficiently expanded in subsections of sensors and devices installations in the following sections. 

## 3. Soil Suction Measured by High Precision Tensiometers

### 3.1. Sensor Functional Mechanism

Since tensiometers were developed earlier by Richards and Weeks [9] and Klute and Gardner [5], it has been applied to measure unsaturated soil water tension or soil suction for an extensive period. The most frequently used tensiometer is the hydraulic connective tensiometer [1,2,9,65], which has similar functionality to a hanging column method (butcher funnel) [38,48,49]. The only difference is that the tensiometer adopts a small ceramic tip intimately contacting ambient soil rather than a complete ceramic plate under the soil specimen at the representative elementary volume (REV) scale. With a high suction yielded in the tested ground, the vertical difference between the ceramic tip and hydraulic head red from the hydraulic connective manometer brings accurate readings of soil suction. Instead of presetting a REV-scale soil specimen on a ceramic plate sealed in the hanging column, exclusively allowing laboratory test, the tensiometer has advantages in applying in both laboratory and field for instantaneous detection of soil suction. As the manometer needs vertical space to read the soil suction head [9,66], it was later replaced by a mechanical pressure gauge to reduce sensor sizes [1,5], followed by the newly developed electrical tensiometers (e.g., the T5© tensiometer produced by UMS [65] GmbH D-81389 München Gmunderstr. 37, München, Germany). Those tensiometers can instantaneously transfer the detected soil suction back to the data logger and later computer. Usually, those sensors can only function properly under the lower suction ranges (0–−160 kPa) due to a few limitations, including the air entry value (AEV) of ceramic tips between 100 kPa and 500 kPa and the bubbling point of −85 kPa for the water stored in the hydraulic connections between the tip and electrical sensor body, etc. As a result, those sensors can only measure soil suction for sandy soils in a full suction range or natural soils with higher fine content in a lower suction range [5,6,9,51,65].

In order to detect soil suction for clay for a higher range, many studies further developed various types of high suction tensiometers [6]. The tensiometers functioning in other mechanisms (e.g., thermal couple sensors) can also be applied to fine soil. Instead of taking advantage of the hydraulic connectivity, those sensors can estimate high soil suction by detecting the thermal conductivity of the water-saturated ceramic tips when those tips have moisture exchange with ambient clay [7,8,10]. With this technique, there is no limitation on the bubbling point as long as the ceramic tip has a high AEV. The thermal couple sensors respond to soil suction and moisture changes very slowly than the hydraulic connective tensiometers. Thus, it might perfectly suit the finer than coarser soil because of lower permeability for both clay and tips in extreme high AEV (e.g., 1000 kPa or even more). In terms of selecting suction sensors, users have to make a trade-off between suction range and responding time, depending on the tested material and research objectives. Overall, the tensiometer technique has been comprehensively developed to cover a wide suction range for all soil types. As the testing condition for all sensors was constrained to a column of uniform sand, the high suction tensiometer is beyond the scope of this work and so will not be further expanded.

**Figure 4:**
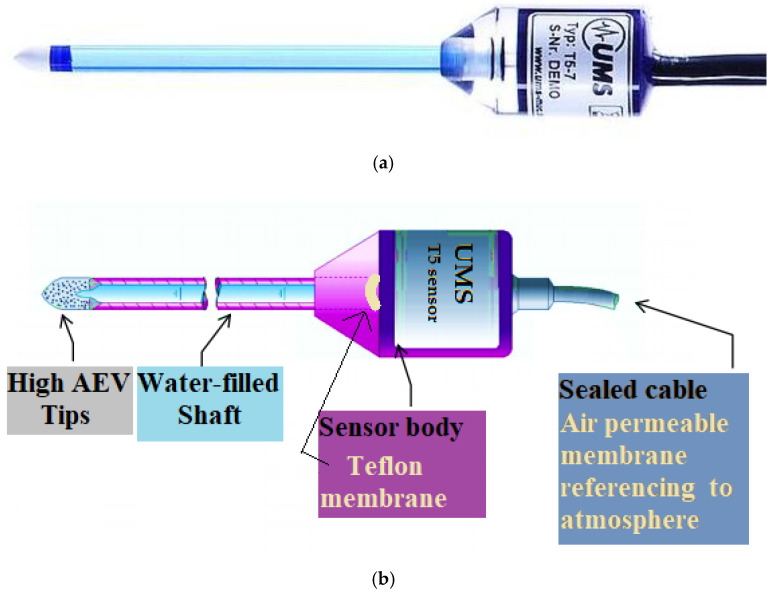
The hydraulic connective tensiometer [65]: (**a**) T5 sensor; (**b**) an illustration of a T5 sensor.

Figure 4a,b separately demonstrate the T5 tensiometer and a schematic description of sensor composition. The T5 tensiometer (UMS) is a soil suction sensor used for measuring negative and positive water pressure from –85 to + 100 kPa with a precision of ±0.5 kPa. Due to the ceramic cup made of Al_2_O_3_ sinter in a minimum size (diameter of 5 mm and length of 6 mm) yielding higher permeability, T5 sensors can achieve a fast and accurate response within 5 s [65]. Figure 4b shows the ceramic tip mounted on a water-filled acrylic shaft (length of 5–20 cm selected depending on inserting depths), screwed into the sensor body (diameter of 20 mm). A Teflon semipermeable membrane (air permeable) in the sensor body varies with hydraulic pressure in the water-filled shaft in order to send signal changes back to a datalogger and then be saved into a digital computer. According to the manual from UMS [65], the transformation between voltage signal and water pressure is given as 85–−100 mV to −85–100 kPa in −1 mV per 1 kPa, and the testing aqueous condition has to be satisfied within pH 3–10. Additionally, the osmotic effect caused by the saline solution will lead to a short delay of response with an overestimation of 1 kPa [65].

### 3.2. Sensor Water-Refilling and Assembling Approaches

**Figure 5:**
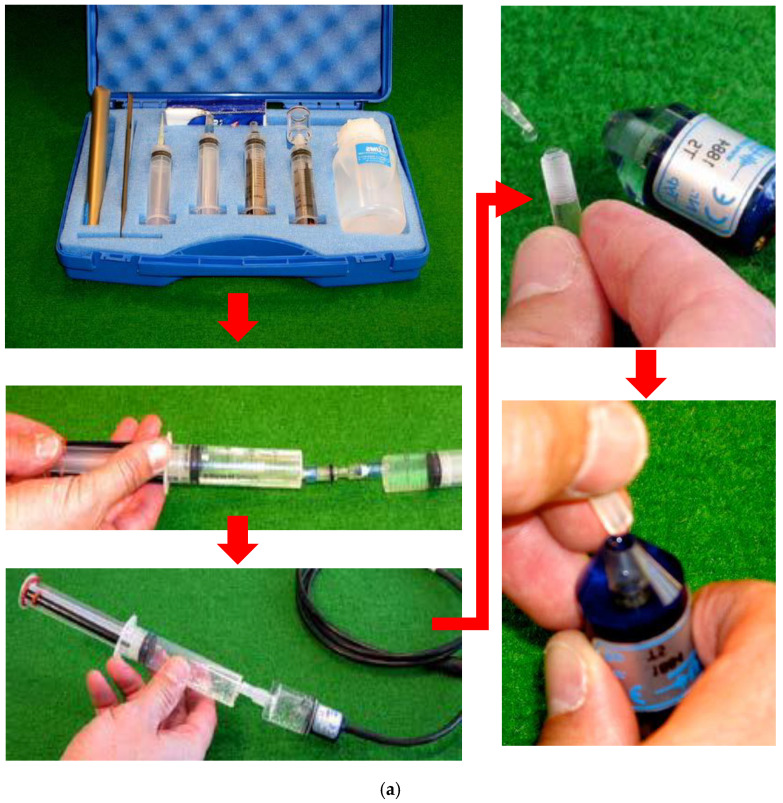

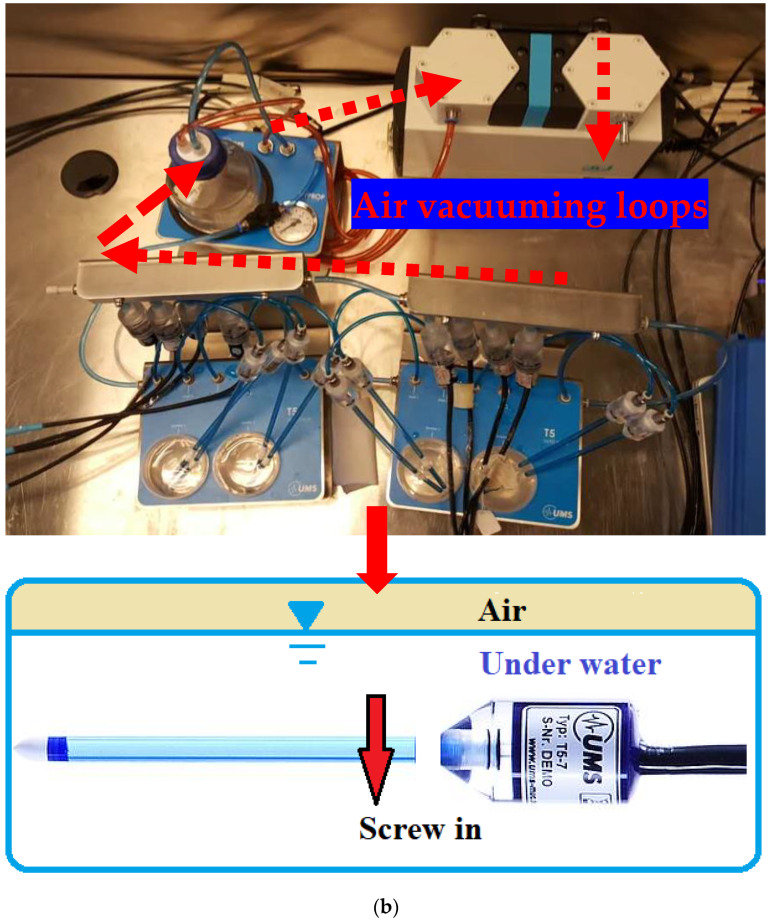
The methods for refilling deaerated water into blue shafts and sensor bodies: (**a**) the manual refilling toolbox-T5 set with a standard operating procedure for T5 refilling and assembly [65]; (**b**) the automatically refilling kit consisting of a white electrical vacuuming pump connected to a water filtering safety bottle with red pipe, chained to two refilling racks and an innovative assembly [36].

Due to the need for a water connection between the membrane in the sensor body and the ceramic tip on the shaft, deaerated water has to be filled into both shaft and sensor body using a manual refilling toolbox-T5 set. Figure 5a briefly shows the standard T5 manual refilling procedure recommended by UMS [65]. In brief, two sensor parts should be separately connected to water-saturated syringes. Then, through a vacuuming process, the deionized water in syringes will be filled into both the shaft through the ceramic tip and sensor body, consequently achieving the deaerated water conditions after two hours of vacuuming [65]. Once fully filled in under deaerated water conditions, both parts can be connected together. It is crucial to be noted that, for ensuring no bubble trapping during connection, deaerated water has to be dripped on both the water-saturated shaft and sensor body before screwing the shaft into the sensor tightly. In general, many previous studies were carried out with this water refilling process, which succeeded many times [45,51,67].

Nevertheless, as each refilling toolbox can only serve each sensor for at least 2.5 h (including 2 h of vacuuming, extra 30 min for refilling and connection operations), it will totally cost several days to fill up 20–30 sensors if and only if none of each manual refilling operation fails. Additionally, during such prolonged refilling work, long term sensor storage issues will potentially undermine the reliability of utilization. Therefore, there is an urgent need for automatic refilling devices to refill sensors massively and simultaneously by mechanical vacuuming than the manual one. Moreover, with such a mechanical pump running for over 48 h, it is possible to generate more reliable bubbly free water for refilling sensors. 

With this motivation, the automatically refilling kit was newly developed by UMS [65]. As shown in Figure 5b, all shafts and sensor bodies should be detached from each other and mounted to the refilling racks. Two refilling racks enable automatically refilling eight sensors simultaneously when they are connected together, directing to the vacuuming pump through a water-filtering safety bottle. With deionized water filled into connectors on each rack, since the vacuum is turned on, water will be sucked into both shaft and sensor body, followed by degassing down to −1 bar (−1 atm = −101 kPa). Many gas bubbles will be formed inside the transparent acrylic sensor body and shafts during this degassing process, so manual flicking operations are highly recommended to be performed frequently within 48 h in order to remove trapped bubbles, similar to knocking trapped bubbles out of the outflow standpipes for a standard pressure plate test [38]. Additionally, it should be noted that when the water pump is switched off, trapped bubbles will be dissolved back into the water if those bubbles are not totally removed. With the measured soil suction increasing, sensors filled with imperfect deaerated water will cause bubbles to form and reduce the shaft’s hydraulic connectivity, consequently delaying and even falsifying the readings. After degassing water for two days, the shaft and sensor can be reconnected together under the deaerated water (see Figure 5b) as an integrated T5 tensiometer. Due to degassing water down to −1 bar and reassembling sensors under the deaerated water, there will be perfectly bubble-free conditions inside each sensor compared to the manual refilling. Additionally, it will not read incorrect soil suction due to gas nucleation and boiling (bubble formed) under low water pressure, unless soil suction is over the AEV of the ceramic tip.

Last but not least, it should also be noted that the air could penetrate into the shaft through the screwing gap between the shaft and sensor under high soil suctions if the shaft is not tightly screwed into the sensor. If this happens, UMS [65] should be the first contact to order a re-machining of the thread on each shaft at fault. The supplier can perfectly repair any shaft thread shrinkage and damage as they have the full capability to machine the dimension of these threads in high precision, meeting the gas-sealing standard. A temporary fix by wrapping a thin layer of thread tap might tight the screwing well but have unobservable gaps resulting in gas penetration.

### 3.3. Sensor Installation Procedure

The T5 sensors can be inserted directly into the soil if relatively soft ground. However, supposing that densely packed sandy soil and hardened clay are encountered, it could be inserted after digging a hole that perfectly fits the sensor shaft using a UMS tensiometer T5-auger kit (TBT 5), shown on the right of the refilling toolbox in Figure 5a. As for disturbed and remoulded specimens in the laboratory, all sensors were priorly inserted into the soil column when fabricating saturated soil specimens in columns. Therefore, each sensor should be horizontally inserted into each column before the water table runs over each inserting point marked as the round cross in Figure 3b during a sample filling process.

**Figure 6:**
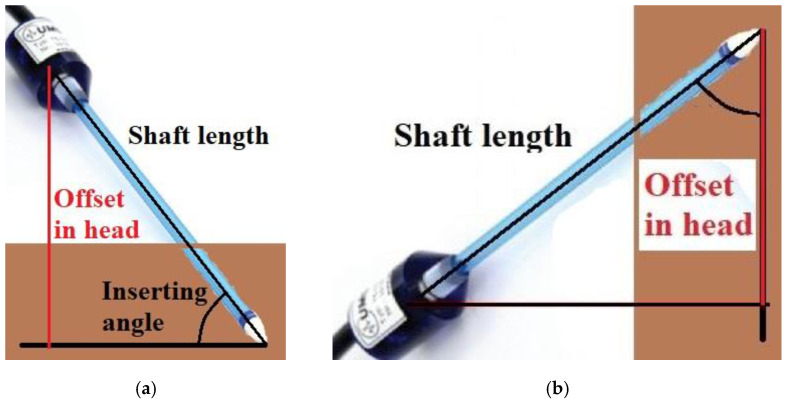
An illustration of the offset caused by inclined insertion: (**a**) the actual pressure head = the reading pressure head + the shaft length × the sine of the inserting angle; (**b**) the actual pressure head = the reading pressure head–the shaft length × the cosine of the inserting angle [65].

The T5 sensors are usually installed on the testing apparatus either horizontally or non-horizontally. When the sensor is inserted horizontally into any soil, there are no hydraulic head differences between its ceramic tip and membrane in the sensor body. Therefore, there is no need for any offsetting adjustment in this condition, which is the case encountered in this work. However, this is not the most available scenario, particularly when the measuring zone does not allow horizontal insertion on a vertically cutting off cross-section in situ. So, offsetting adjustments must be carried out when the sensor is inclinedly inserted into the soil within a specific angle from flat ground to the sensor shaft (90° for vertical insertion). An illustration of offset adjustment is shown in Figure 6a, where the actual pressure head should be the reading pressure head plus the shaft length timed by the sine of the inserting angle. The minus inserting angle is rarely seen for practice. If it accidentally occurs (see Figure 6b), the actual pressure head should be the reading pressure head deducted by the shaft length timed by the cosine of the inserting angle.

### 3.4. Simultaneous Data-Logging Configurations

**Figure 7:**
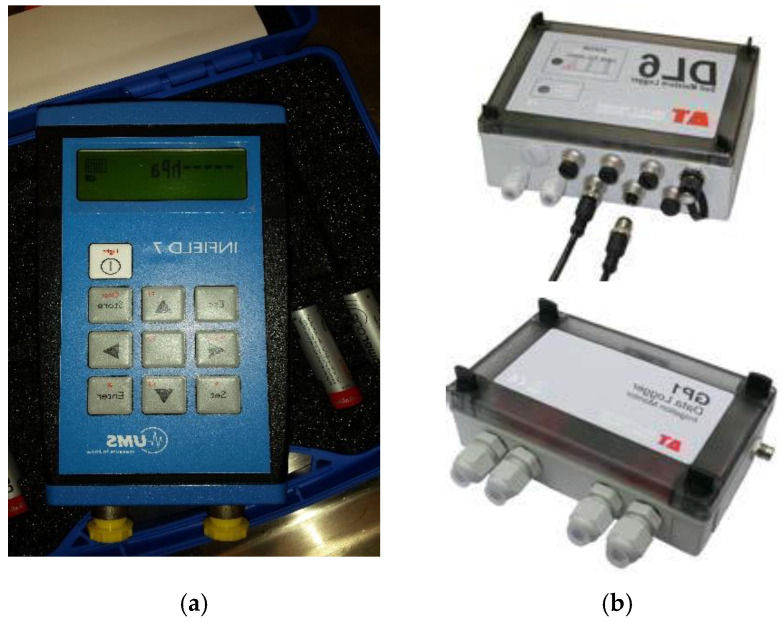

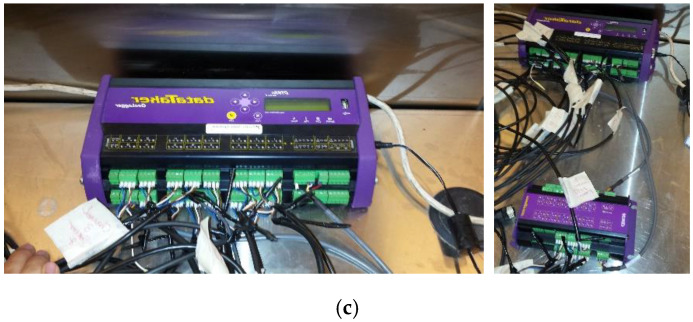
Three data logging systems for T5 tensiometers: (**a**) the Infield 7 hand reader provided by UMS [65]; (**b**) the 6-channel DL6-te and dual-channel GP1-te data loggers provided by UMS [65]; (**c**) the 18-channel DT85g Series 3 DataTaker GeoLogger linked with the 20-channel CEM20 channel expansion board, allowing a maximum number of 37 analogue channels connected to sensors [68].

Soil suction measured by the electrical sensor cannot be directly read without a digital reader or computer. So UMS [65] provides two options: the Infield 7 (UMS) hand reader or DL6-te and GP1-te tensiometer loggers (UMS), separately shown in Figure 7a,b. Those logging systems can directly output the soil suction without calibration between voltage and water pressure because they are custom designed and manufactured to serve UMS sensors. In addition, all T5 sensor cables have a four-pin connector that can be directly connected to those devices without any connector modification.

This series of soil column tests required a total of 24 channels for four soil columns (6 channels to 6 T5 sensors for each soil column). Due to a shortage of enough channels in the 6-channel DL6-te data loggers and a cost-effective trade-off between buying four such loggers and seeking a more cost-effective logging solution, the custom-made loggers were replaced by a non-custom-made one, the 18-channel DT85G Series 3 DataTaker GeoLogger (Pacific Data©) linked with the 20-channel CEM20 channel expansion board, allowing a maximum number of 37 analogue channels connected to sensors. The recording temporal resolution was set at 30 s. With this logger, the total procurement budget can be dramatically minimized, and its reliability has been verified well in many field data logging works [68].

### 3.5. Post Signal and Data Processing

**Figure 8:**
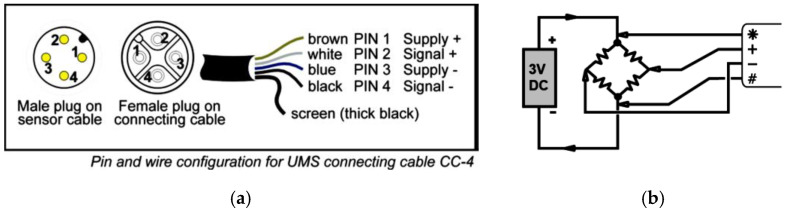

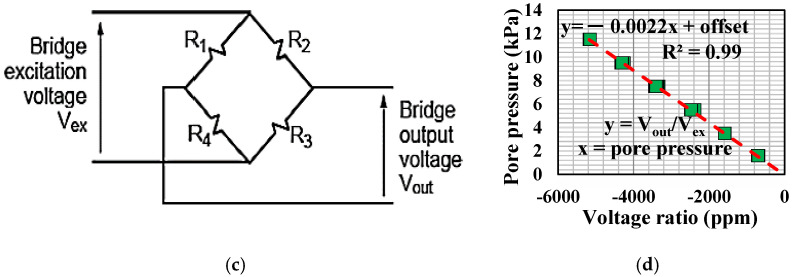
The connector configuration between the T5 sensor and logger analogue channel and corresponding signal processing between the voltage in/outputs from the T5 Wheatstone circuit and pore pressure: (**a**) the T5 sensor connector design; (**b**) the analogue channel on GeoLogger compatible with the Wheatstone circuit with an additional 3 V power supply; (**c**) the voltage in/outputs of Wheatstone circuit; (**d**) the linear calibration between voltage output and pore pressure [16,65,68].

The only effort to be spent is to modify the T5 sensor four-pin connectors to be compatible with the four-pin analogue channels on GeoLogger (see Figure 8a,b) and conduct a specific calibration between output voltage signal and measured water pressure (see Figure 8c,d). In addition, this data logger also needs an experienced user to configure the data logging program using either its graphical user interface or command interface in coding style. Furthermore, users have to be very familiar with the electric circuits of the T5 sensor and the data logging code in the program. According to the T5 manual from UMS [65], the sensor electric circuit is a Wheatstone bridge circuit (see Figure 8c). Therefore, in order to carry out signal logging from T5 sensors using the coding interface of GeoLogger, the 3 V electrical power-supplied output is required for such a circuit by using the logger-specific code “BGI (4 W, “sensor labelling number”)” or “BGI (“sensor labelling number”)”, respectively. The “4 W” and without “4 W” separately indicate the current-excited and voltage-excited Wheatstone bridge circuits. Both options can be applied to extracting voltage signals from the sensor to the datalogger. All data will be saved in the logger, which has 2 GBs of data storage and cannot be directly sent to a computer. Hence, users have to manually extract data from the logger using the graphical interface every 1–2 days in case new data overwrite the old data in such a limited data storage.

Figure 8d shows the linear calibration between the output signal (bridge excitation voltage over bridge output voltage, V_out_/V_ex_) and soil pore water pressure. A slope of –0.0022 could be determined between the voltage ratio and pore pressure with an offset, which has nothing to do with the previously elaborated sensor installation. This offset only matters with the aging membrane in the sensor body. Due to the aging issue, the membrane might not function as elastic as it used to be and therefore falsify the reading with a bias. However, it is possible to measure the pore pressure variation accurately by adjusting such a bias to zero. This bias can be determined as the interception (offset) of the linear calibration in Figure 8d. 

**Figure 9:**
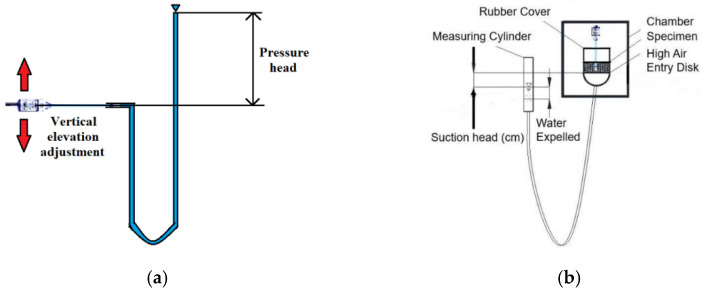
Two methods for calibrating T5 sensor voltage ratio output against actual soil water pore pressure: (**a**) the positive pressure–voltage ratio calibration using a U-shape manometer; (**b**) the negative pressure–voltage ratio calibration using the hanging column method with a suitable soil preset.

There are mainly two methods to calibrate the T5 sensors when adapted to a non-custom data logger or need to determine the membrane aging-induced offset. Figure 9a shows the linear calibration between voltage ratio and positive hydraulic pressure. With a U-shape manometer, the hydraulic head difference can be correspondingly subjected to a voltage ratio recorded by GeoLogger in order to plot the linear regression in Figure 8d. Moreover, this sensor can also be calibrated by a hanging column method, as shown in Figure 9b. The soil suction versus voltage ratio can also be given via inserting a T5 sensor into the centre of the hanging column test. By adjusting the vertical elevation of the measuring cylinder, a soil suction can be applied after the water table reaches equilibrium every two days later. Then, the voltage ratio and soil suction head could be both recorded for a linear calibration. Basically, both calibrations should provide the exact same linear regression equation with an R-square of 99%.

**Figure 10:**
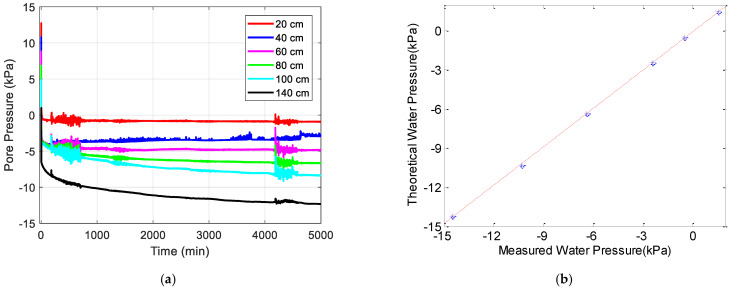
The output from the logging system with the T5 sensors: (**a**) the nonequilibrium and equilibrium soil suction measured by T5 tensiometers; (**b**) the validation of measured pore water pressure against theoretical water pressure above groundwater table.

Eventually, all voltage signals recorded by the data logger can be transferred to actual pore water pressure in positive and negative ranges using those calibrations. The negative pore water pressure is the so-defined equilibrium soil suction. An example of the dynamic response of pore pressure under one-step outflow for the 30/60 sand column is presented in Figure 10a. In Figure 10b, the final pore water pressure (the final equilibrium soil suction) is compared to the negative pore-water pressure (the theoretical soil suction), which is calculated as the water density timed by the vertical distance from the groundwater table.

## 4. Soil Moisture Content Measured by Spatial Time-Domain Reflectometry Technique

### 4.1. Sensor Functional Mechanism

**Figure 11:**
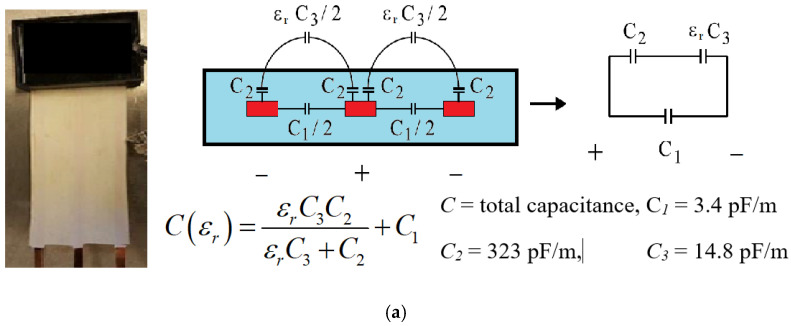

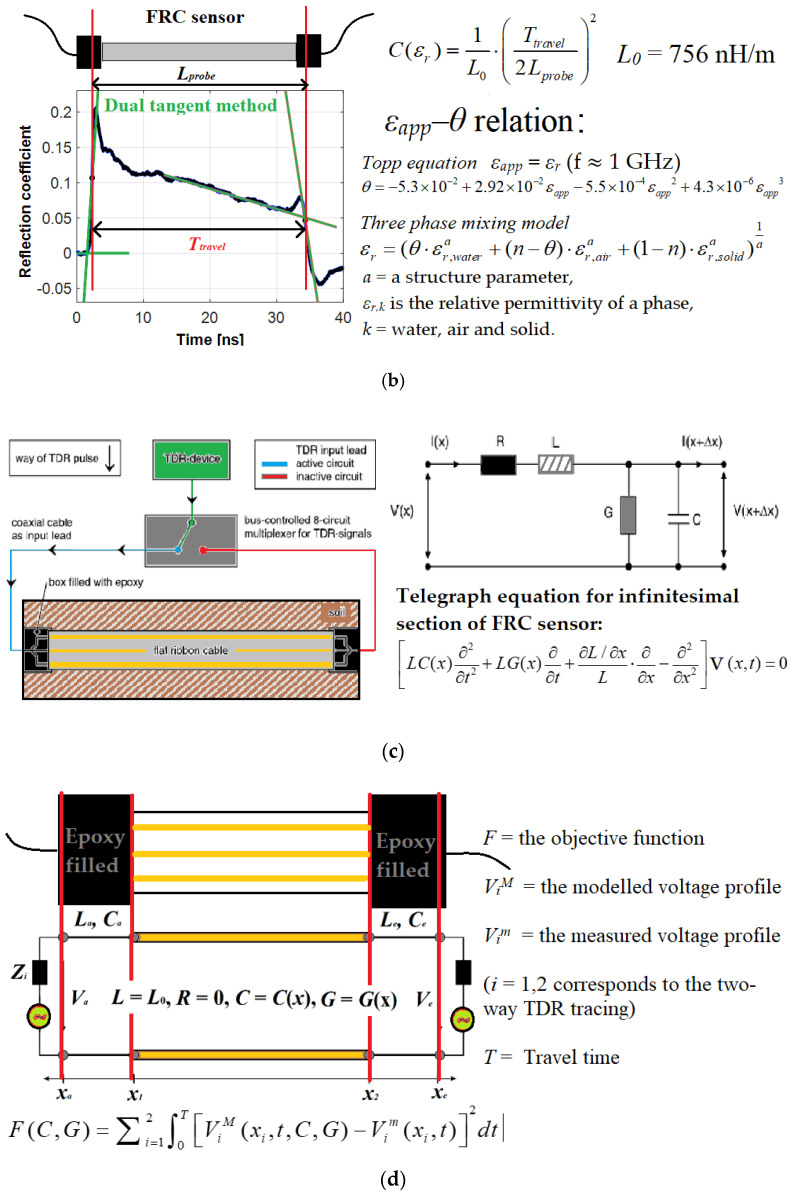
The comprehensive illustration of the spatial TDR technique with the FRC sensor design: (**a**) the FRC sensor design and capacitance model; (**b**) the conventional TDR application using the FRC sensor; (**c**) the illustration of two-way TDR tracing and telegraph equation modelling FRC sensor as transmission lines; (**d**) the spatial TDR inversion analysis with an illustration of the optimization objective function between the measured and modelled TDR waveforms [11,15,69,70].

The spatial time domain reflectometry (spatial TDR) is a unique TDR technique, incorporating the conventional TDR with a flat ribbon cable (FRC) sensor (see Figure 11a,b) designed by Huebner, Schlaeger, Becker, Scheuermann, Brandelik, Schaedel and Schuhmann [11] and a fast TDR inversion technique (see Figure 11c,d) developed by Schlaeger [69] under a frequency (*f*) assumption (*f* < 10^4^ Hz) [11,15], within which there are constant inductance (*L*) and resistance (*R*), and frequency-independent conductance (*G*) and capacitance (*C*) profiles along the sensor waveguide. To expand this highly concise definition of the spatial TDR to detail, its functional mechanism can be introduced in Figure 1 with the following illustrations.

Similarly to the conventional three-pin TDR sensor, the new designed FRC sensor also has three copper wires as the waveguide. Still, they are insulated by a polyethylene cover to prevent direct contact with ambient testing environments. Due to such a thin layer cover, the measurement of apparent dielectric permittivity (*ε_app_ = ε_r_* (*f* = 1 GHz), when dielectric permittivity at the frequency of around 1 GHz) of ambient environments is no longer the *ε_app_* of surrounding soil but a combination of both soil and isolation cover. To distinguish the *ε_r_* of the surrounding earth from the *ε_r_* of ambient environments, Huebner, Schlaeger, Becker, Scheuermann, Brandelik, Schaedel and Schuhmann [11] developed a capacitance model for the cross-section of FRC sensor as illustrated in Figure 11a, where the capacitances (*C*_1_ and *C*_2_) in isolation and the soil capacitance (*C*_3_) have been clearly defined.

The total capacitance, *C*(*ε_r_*), of ambient environments could be determined based on the conventional TDR technique. As illustrated in Figure 11b, with known sensor inductance, *L_0_* = 756 nH/m [15], and waveguide length (*L_probe_*), the *C*(*ε_r_*) could be calculated with only the travel time (*T_travel_*) predetermined by the dual tangent method on the measured TDR waveform [71]. Since the *C*(*ε_r_*) is determined, it is then possible to inversely calculate the *ε_app_* = *ε_r_* of ambient earth according to Figure 11a. With any reliable apparent permittivity-soil volumetric moisture content (*ε_app_–θ*) calibrations or empirical equations [72,73,74], the point-wise moisture measurement can finally be achieved like the conventional TDR technique but with the FRC sensor specifically designed for measuring moisture profile along a transect.

To further extend the capability from the point-wisely standard TDR to the spatial TDR capable of measuring the *θ* profile, the sensor could be conceptually seen as a transmission line that the telegraph equations can model in Figure 11c [11]. As a result, it is expected that it will be possible to finally inversely determine the *C* profile as closely as possible to the actual *C* profile with a forward modelling telegraph equation based on an initial guess of the *C* profile and backward updating the *C* profile based on measured TDR waveforms through an optimization algorithm, as illustrated in Figure 11d. Such challenging work was achieved with a few assumptions on constant inductance (*L = L_0_*) and resistance (*R* = 0), and frequency-independent *C(x)* and *G(x)* profiles along the sensor waveguide (see Figure 11d) [11,15]. Based on those assumptions, Schlaeger [69] developed a numerical solution to the telegraph equation using a finite difference scheme coupled with an optimization algorithm minimizing the difference between forward modelled and measured TDR waveforms using a conjugate gradient method. When a global optimum is settled, the input of the *C*(*x*) profile could be identified as the reconstructed *C*(*x*) profile for later sequential determinations of *ε_r_*(*x*) and *θ*(*x*) profiles based on Figure 11a,b. Moreover, as there are two unknown profiles, not only *C(x)* but also *G(x)*, the two-way TDR tracing, sending and later receiving TDR waveform from both ends of a FRC sensor by switching connection in multiplexer shown in Figure 11c, needs to be carried out to access two TDR waveforms for the fast TDR inversion analysis. Therefore, the two-way TDR inversion analysis provides both *C* and *G* profiles while only *C(x)* is useful for *θ(x)* determination. 

### 4.2. Sensor Manufacturing Approaches

**Figure 12:**
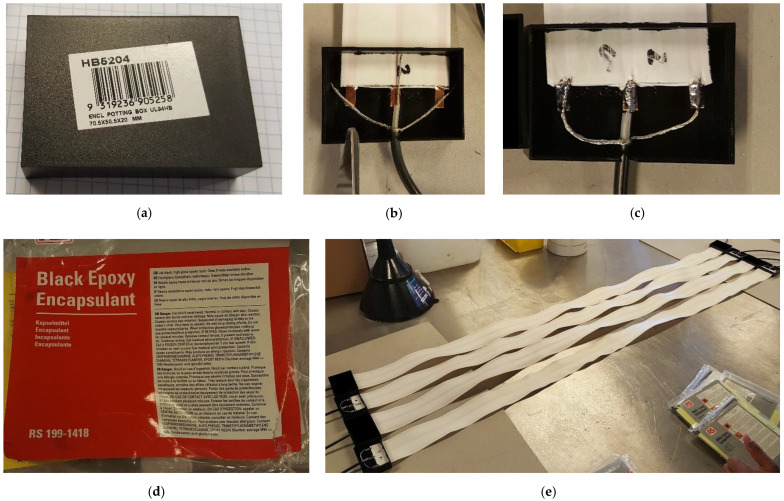
The flat ribbon cable (FRC) sensor manufacture: (**a**) the black terminal box for sealing connections between a coaxial cable and an FRC; (**b**) a demo of the wire configuration between two cables; (**c**) a demo of the coaxial cable soldered to copper wires in the FRC; (**d**) the black epoxy encapsulant applied to seal the terminal box; (**e**) a demo of finished sensors compared to unfinished ones.

The spatial TDR sensor comprises one white flat ribbon cable, both ends of which are separately connected to a coaxial cable and then filled up with epoxy for waterproofing. In brief, the sensor manufacturing procedure can be summarised in the following steps:

(1) Access the white ribbon cables, coaxial cables and black terminal boxes (see Figure 12a,b) from a local retailer dealing in electronic components (e.g., Jaycar, Australia);
(2) Drill a hole and a gap on both sides of the black terminal box;
(3) Separately fit a coaxial cable and white ribbon cable into the box through the hole and gap predrilled (see Figure 12b);
(4) Configure the wires in the coaxial cable to three copper transmission lines in the white ribbon cable as shown in Figure 12b;
(5) Solder the configured wires like Figure 12c;
(6) Seal the holes and gaps between the black box and two fitted-in cables with plasticine in case later the liquid epoxy leaks from those gaps;
(7) Access black epoxy (see Figure 12d) and fill liquid epoxy into the half-opened terminal boxes to ensure a perfect waterproof sealing;
(8) Tap terminal boxes filled with liquid epoxy to ensure no trapped air inside the boxes;
(9) Wait for the liquid epoxy to solidify until the sensor is successfully manufactured, as shown in Figure 12e.

It is worth noting that the lengths of a ribbon cable inserted into both terminal boxes should be precisely equal because it is identified as part of the symmetric sensor beginning. In addition, the capacitance of the used epoxy should be specifically tested because both epoxy-covered terminals should also be counted into the capacitance and dielectric permittivity profiles for the fast TDR inversion analysis illustrated in Figure 11d. 

### 4.3. Sensor Installation Procedure

**Figure 13:**
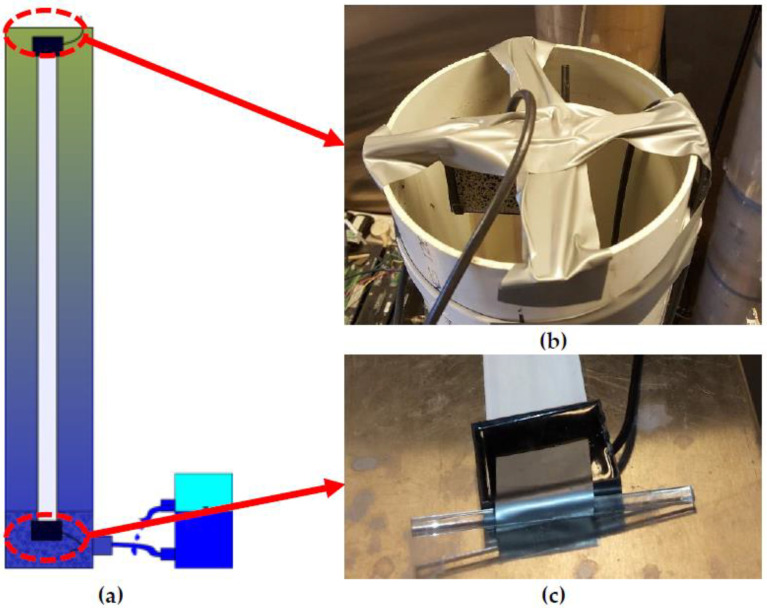
The central-located sensor installation method: (**a**) a demo of a central-located FRC in a soil column and the sensor bottom end is fixed by a gravel filter; (**b**) a cross holder fixing the sensor top end at the empty column top; (**c**) a rod attached to the sensor bottom end embedded in the filter.

As illustrated in Figure 3 and Figure 13a, in order to accurately measure the soil moisture content profile, the FRC sensors ought to be embedded into the centre of soil columns without any air gap between the sensor body and the surrounding soil. This is because the air gap between sensor and soil will lead to significant measuring errors, which have been specifically analysed by an electromagnetic finite element simulation study by Wagner et al. [75].

In fact, there are two main methods for sensor installation. The most common one is the central-located method. Figure 13a clearly illustrates how the FRC sensor is centrally prelocated in an empty column before specimen fabrication. The bottom end of a sensor is attached to an acrylic rod (see Figure 13c), in order to be fixed by a gravel filter at the column bottom. The sensor top end is attached to a cross holder, which right caps on the column top. With those two holders on both the column top and bottom, the entire FRC sensor can be stretched straight without any bending and twisting, because the geometric distortion of the sensor body causes inductance variation, further undermining the reliability of assumptions in the TDR inversion technique. Then, the specimen fabrication can be carried out to achieve a relative homogeneous soil profile in Figure 2b by following the sample loading procedure shown in Figure 2a. With this soil profile fabrication method, not only are the constant density and porosity achievable but the air gaps between the ambient soil and FRC sensor could also be avoided if there is no shrinkage of the specimen after a drying process. Hence, soil column tests still seem not highly applicable to soil samples with high clay content.

The other method was developed by Scheuermann, Montenegro and Bieberstein [45], in which one side of the FRC sensor was tightly contacted with an acrylic column and the other side was covered by the tested sand specimen. Under such a sensor installation condition, the conventional sensor calibration will no longer be valid as one side of the sensor is not measuring soil but the solid acrylic material. Therefore, a very specific calibration between dielectric permittivity and soil moisture content needs to be carried out for such a condition rather than directly applying any empirical equations and the three-phase mixing model previously mentioned in Figure 11b. This method is not only applicable for a column test in the laboratory but also to the field conditions when high clay content-induced contact loss between the FRC sensor and ambient earth profile. The FRC sensor can be suppressed to a vertical earth profile with excellent contact by continuously inflating a thick rubber balloon inside a borehole, so shrinkage around the borehole cannot jeopardize the measurement accuracy. However, this method is not universal for all cases. For example, when clay content is higher enough to raise the frequency dependence of dielectric permittivity (*f* < 10^4^ Hz), the fundamental assumptions for the TDR inversion analysis are not available, no matter what specific sensor installation and calibration could be performed.

### 4.4. Simultaneous Data-Logging Configurations

Figure 3a already shows four FRC sensors prelocated in the centre of four sand columns. Additionally, as the two-way TDR tracing demonstrated in Figure 11c, each FRC sensor has two coaxial cables, demanding two channels connected to a TDR device. In total, there are eight channels in need of TDR pulse generating and receiving. However, a single TDR device, e.g., TDR 100 provided by Campbell Scientific©, only has only a single channel. Therefore, for conducting two-way TDR tracing for four sensors almost simultaneously, the TDR 100 was connected to an SDM X50 multiplexer and CR1000 data logger, both provided by Campbell Scientific© as well. Each multiplexer can provide eight channels that perfectly suit the need of this work. The conventional TDR technique requires manually conducting TDR measurements using PC-TDR software developed by Campbell Scientific© with TDR 100 device. In contrast, it is capable of setting up an automatically logging program for eight channels of four sensors in a sequence every minute with the CR 1000 logger and appending software-LoggerNet Ver 4.2, also developed by Campbell Scientific©. 

**Figure 14:**
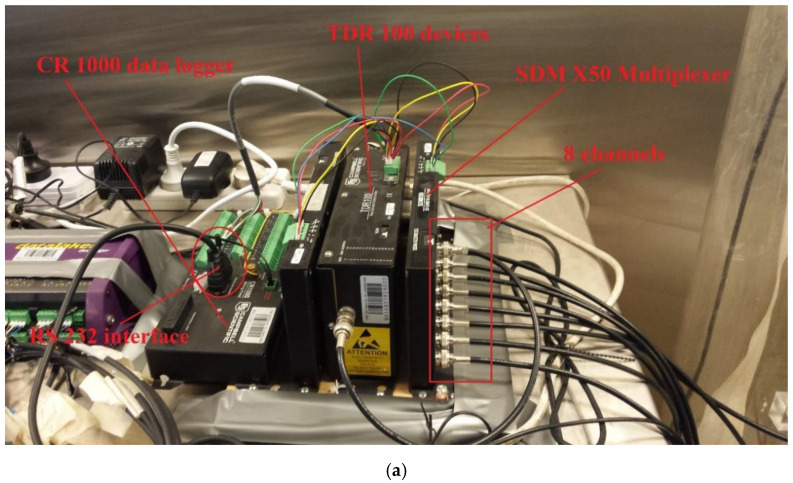

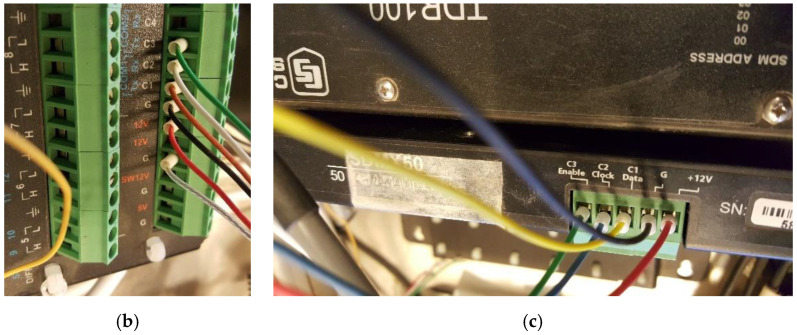
The spatial TDR data logging system: (**a**) the entire data logging system consists of a CR1000 datalogger connected to a TDR 100 pulse generator/receiver and SDM X50 multiplexer having eight channels available for four FRC sensors; (**b**) the data wire configuration on CR1000 data logger; (**c**) the data wire configuration on TDR 100 device and the SDM X50 multiplexer.

A demonstration of this data logging system is shown in Figure 14a. Eight coaxial cables were connected to the multiplexer, which is connected to a TDR 100 pulse generator and receiver by a coaxial cable. Both TDR 100 and SDM X50 multiplexer were configured to the CR 1000 data logger by a series of colourful data wires, as shown in Figure 14b,c. An RS232 to USB in/output interface on the datalogger can be used to communicate with the computer for consistently sending logging programs to the datalogger and transmitting TDR waveforms back to the computer. A ground wire should be configured from the data logger to the ground to avoid signal noises. The twelve-volt electrical power should be consistently supplied to the data logger in order to power both TDR 100 and the multiplexer.

The data logging program in LoggerNet could be configured using the graphic interface in the software. However, such an interface is only applicable to commercial TDR sensors listed in the software. As the FRC sensors were self-made for specific academic purposes, it is not listed in the LoggerNet, so a particular logging schedule had to be programmed by CR Basic language. For example, to call the TDR waveform logging in CR1000, the code “*TDR100 (the data file name, the SDM address, the measuring option, the multiplexer/probe selection, the TDR wave averaging times, the propagation velocity, the data point number per TDR waveform, the coaxial cable length, the measuring window length, the probe length, the probe offset, the multiplexer number, the overall offset)*” should be executed. Using the graphical interface, other logging schedules could be first generated and then changed to suit specific time schedules by following the Basic language. The physical settings of FRC sensors in this work are presented in Table 2. All those settings are selected based on the FRC sensor design of 150 cm long with 300 cm coaxial cables connected to both epoxy terminals. Other settings in the code “*TDR100*” that have not been mentioned in Table 2 should adopt the default settings if the TDR 100 and multiplexer configuring panel are in the default settings. Corresponding changes should be made if those configurations are adjusted for connecting with more than one multiplexer. Moreover, the recording temporal resolution was set at 30 s.

### 4.5. Post Signal and Data Processing

#### 4.5.1. The Multiplexer-Induced Underestimation of Reflection in TDR Waveforms

**Figure 15:**
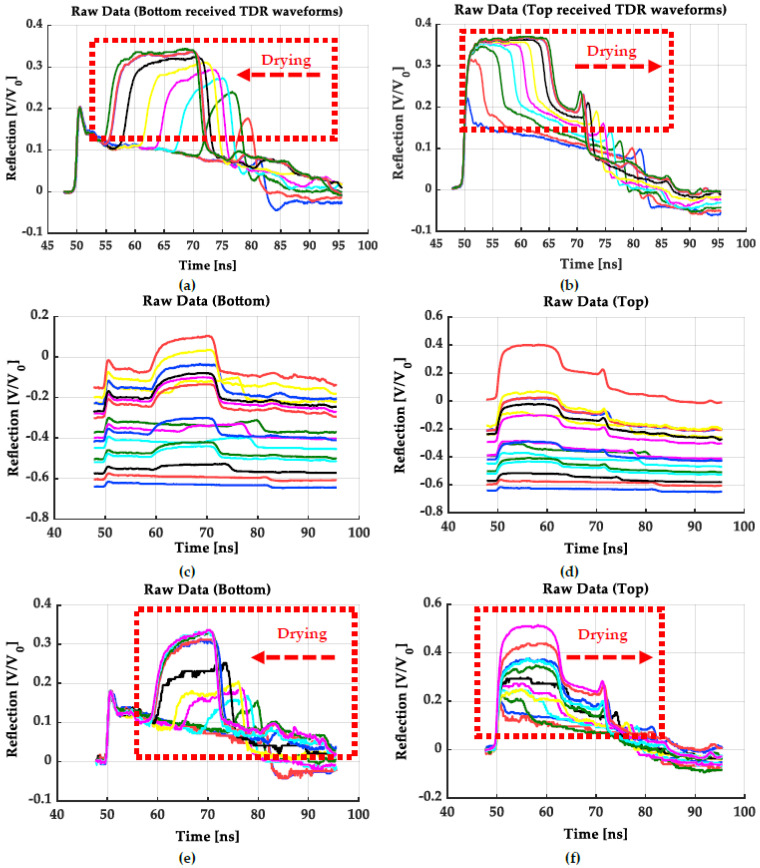
The TDR waveforms detected through the SDM X50 multiplexer: (**a**) a successful demo of TDR waveforms detected at the bottom end of an FRC sensor; (**b**) a successful demo of TDR waveforms detected at the top end of an FRC sensor; (**c**) a failure demo of TDR waveforms detected at the bottom end with the multiplexer-induced underestimation of reflection coefficients; (**d**) a failure demo of TDR waveforms detected at the top end with the multiplexer-induced underestimation of reflection coefficients; (**e**) a demo of rescued TDR waveforms detected at the bottom end after applying the correcting step introduced in this work; (**f**) a demo of rescued TDR waveforms detected at the top end after applying the correcting step.

The outputs from the data logging system mentioned above are a series of TDR waveforms in the temporal sequence received from both ends of an FRC sensor. Many very successful instances have been demonstrated in previous literature of many studies [11,15,16,19,20,21,45,69,71,76,77]. Here, a perfect example is shown in Figure 15a,b for demonstrating TDR waveforms detected at the bottom and top of a soil column during a one-step outflow test. The second reflection point decreases with the reflection coefficient (V/V_0_, the receiving voltage by the pulsing voltage) increasing during the ongoing drainage process. Such a perfect TDR waveform data logging is attributed to the proper functioning SDM X50 multiplexer. Additionally, all those TDR waveforms can be directly applied for the TDR inversion analysis.

However, the multiplexer is not always reliable and might cause unreliable TDR waveforms because of multiplexer-induced interference [26] and even delivers total noises without any valuable and extractable information. Under such circumstances, the detected TDR waveforms presented in Figure 15c,d are not directly applicable to the TDR inversion analysis. However, it is still available for a conventional TDR analysis because the dual-tangent method is applicable as long as reflection varies at two inflection points, and incorrect reflection coefficients will not adversely influence the determination of the TDR travel time by this method. 

Therefore, a correction step that has never been reported before is succinctly introduced here to rescue those TDR waveforms for the TDR inversion analysis. First, a TDR waveform from the bottom end in the saturated zone should be detected by directly connecting to the TDR 100 device without the multiplexer. Such a TDR waveform will deliver a correct TDR waveform without initial reflection deviation and incorrect reflection (see Figure 15c,d). Then, the reflection coefficient of the first peak could be recorded as a referencing point for later correction. Finally, all TDR waveforms detected through the multiplexer should be divided by their original first peak values on the *y*-axis and then timed by the referencing reflection coefficient determined in the previous step. The TDR waveforms in Figure 15c,d can be eventually reconstructed, as shown in Figure 15e,f, which are applicable to the TDR inversion analysis as same as Figure 15a,b.

The fast TDR inversion analysis developed by Schlaeger [69] overcame the multiplexer-induced initial reflection deviation and smoothness of TDR waveforms but has not included an additional correcting step for the multiplexer-induced incorrect reflection coefficients. Without this correction step, the optimization will fail to fit the simulated waveforms into the measured waveforms as the measured ones are too flat with multiplexer-induced underestimation of reflection coefficients. Here, this technical note can improve the applicability of the TDR inversion analysis because the two-way TDR tracing requires a minimum of two available channels for an FRC sensor, which can only be conducted with a multiplexer.

#### 4.5.2. The Settings of the Fast TDR Inversion Analysis

Since this correcting step rescued the TDR waveforms, all waveforms could be inversely analysed using the forward modelling telegraph equation and optimization illustrated in Figure 11c,d. However, prior to conducting the inversion analysis, a very specific and detailed list of parameters regarding sensor design should be carefully assigned along all transmission lines and the sensor isolation material. Table 3 lists those sensor parameters in detail, and each parameter can be checked in Figure 11 accordingly. With all those parameters available, the inversion analysis can finally be carried out by a computer program developed by Schlaeger [69], which was coded in earlier versions of Matlab before 2013 (Mathworks. Inc., Portola Valley, CA, USA).

#### 4.5.3. The Performance of the Fast TDR Inversion Analysis

**Figure 16:**
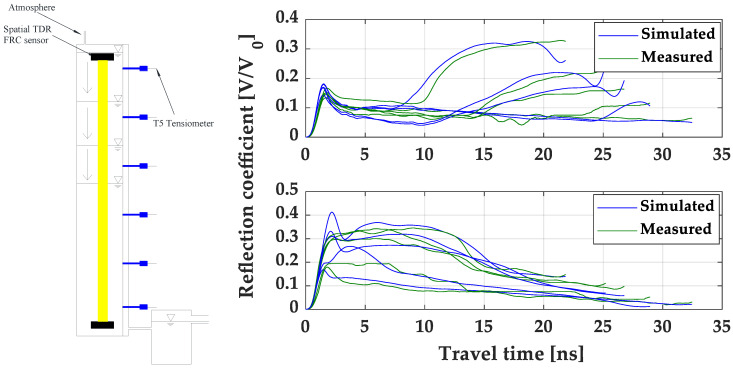
A demonstration of TDR waveforms reconstruction using the fast TDR inversion analysis: the top subplot shows the two-way TDR tracing from the sensor bottom end, and the bottom subplot shows the two-way TDR tracing from the sensor top end with an illustration of the one-step outflow in the soil column filled with Sebilco (30/60) medium sand provided by Sebilco, Australia.

The spatial TDR inversion analysis is highly dependent on the performance of optimization between the simulated and measured TDR waveforms. For a demonstration of the successful implementation of the inversion algorithm, the TDR reconstruction performance for the selected medium sand column is shown in Figure 16, with a schematic description of the one-step outflow drainage test. Figure 16 presents the TDR waveform variations sent from both ends in terms of the increase in reflection coefficients with the groundwater table dropping downward during an ongoing drainage process, as illustrated on the left of Figure 16. The reduction in travel times between two inflection points corresponds to the decreasing average moisture contents in the soil column. In Figure 16, there are acceptable agreements between the TDR waveforms simulated by the forward modelling telegraph equation and measured by the TDR equipment. The TDR waveforms varying with soil water drainage show that the information in TDR waveforms can be used to extract the dynamic soil moisture content profiles in addition to the point-wise average soil moisture content given by the conventional TDR. Additionally, each simulated TDR waveform was generated and finally accepted without any further improvement of matching after 20–40 iteration steps in the optimization algorithm.

#### 4.5.4. The Outputs of the Fast TDR Inversion Analysis

**Figure 17:**
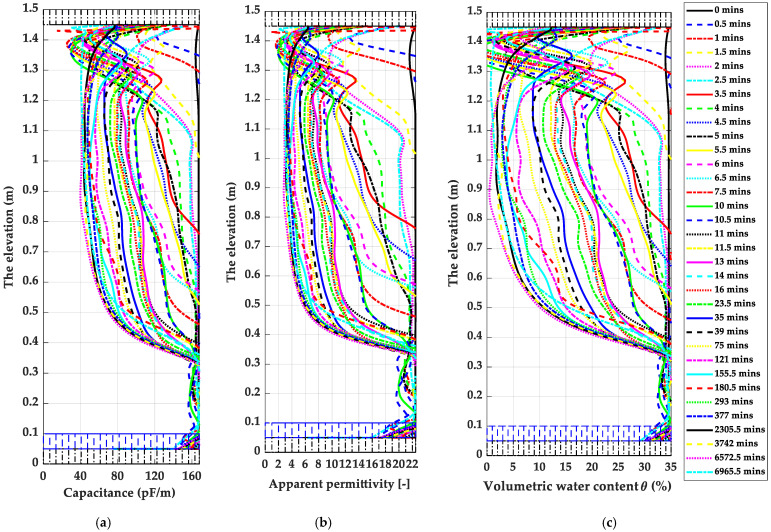
The demonstrations of reconstructed capacitance, apparent permittivity and volumetric moisture profiles (*C(x), ε_app_(x)* and *θ(x)*) for the selected soil column filled with Sebilco 30/60 sand using TDR inversion analysis: (**a**) *C(x)*; (**b**) *ε_app_(x)*; and (**c**) *θ(x)*.

The inputs of total capacitance and conductance profiles *C(x)* and *G(x)* for yielding the best-matched TDR waveforms were inversely determined as the measured total capacitance and conductance profiles of the material mixture covering three copper wires in the FRC sensor, including polyethene waterproofing cover and ambient soil samples. With the capacitance model of the FRC sensor illustrated in Figure 11a, the total capacitance profiles, *C(x)*, can be transferred into the soil apparent permittivity profiles, *ε_app_(x)*. Such an *ε_app_(x)* can be then used to calculate the soil volumetric moisture content profiles, *θ(x)*, using the specific calibrations in Figure 11b. The dynamic profiles of inversely determined *C(x), ε_app_(x)* and *θ(x)* are shown in Figure 17a–c, respectively. Figure 17 demonstrates the success of the spatial TDR technique application. Such a TDR technique enlightens more opportunities for the hydrologist and geotechnical academia to acquire higher temporal resolutions of soil volumetric moisture content in distributions. Furthermore, with an excellent appreciation for developing the TDR inversion analysis, the spatial TDR technique can further investigate scale effects in soil water retention behaviour [35,78,79] and dynamic capillary effects in transient two-phase flow in porous media [4,80,81,82].

## 5. Soil Accumulative Outflow Measured by Digital Bench Scales

### 5.1. Design of Accumulative Outflow Measurement

The in/outflow of a one-dimensional soil column could be measured using versatile methods: (1) the flow rate logging using flowmeters; (2) the accumulative soil moisture discharge logging by applying conventional TDR analysis for an FRC sensor; (3) the weight logging for the entire soil column or outflow tanks using digital bench scales. The flowmeters have successfully been applied to measure in/outflow rates by many single-phase seepage experiments integrated with the TDR sensor technique in previous studies [19,20,21,28,83,84]. All those studies applied a high-precision electromagnetic flowmeter for flow rates logging at higher flow rates, which were generated for a single-phase flow seeping through perfectly spherical glass beads in various sizes under unconsolidated conditions. However, suppose the granular glass beads are replaced by natural soil in a non-spherical shape under consolidated conditions. In that case, the flow rate will apparently be decreased, which could bring challenges to the precision of flowmeters. In addition, with the single-phase shifted to the two-phase seepage, such flow rates could be further reduced. Therefore, flow meters with much higher precision to detect unsaturated soil water flow rates could be out of budget and even not approachable for ordinary geotechnical laboratories. To minimize the budget and maximize cost-performance for an outflow logging system, both the FRC sensors integrated with conventional TDR technique and digital bench scales were adopted instead of extremely high precision electromagnetic flowmeters.

**Figure 18:**
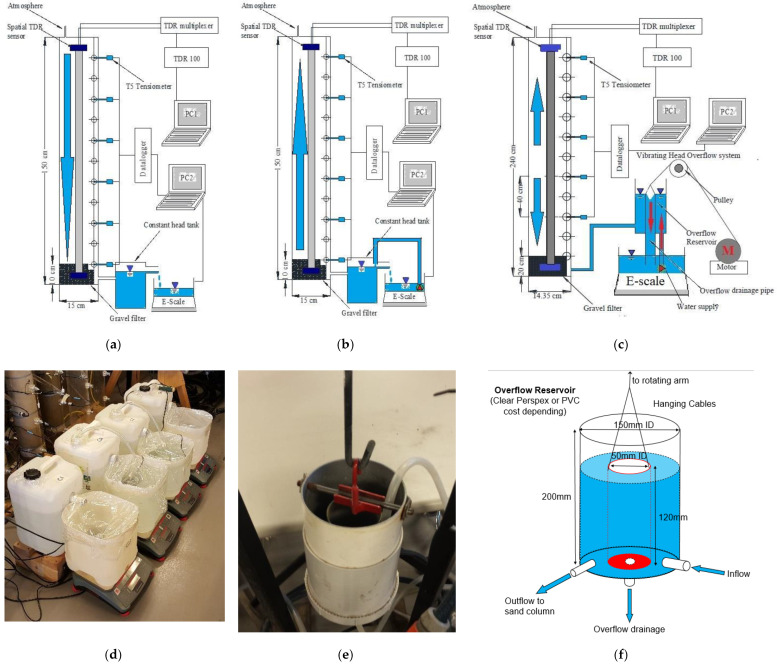
The accumulative in/outflow measurement using the electrical bench scales (E-scale) and spatial TDR sensors: (**a**) the one-step outflow test for drainage; (**b**) the one-step inflow test for spontaneous imbibition; (**c**) the multistep in/outflow test for hysteresis; (**d**) the actual bench scales arrangements for in- and outflow tests; (**e**) a demo of vibrating head overflow (VHO); (**f**) a design of VHO.

Figure 18a–c separately illustrates three designs of the accumulative outflow measurement by the spatial TDR sensors and electrical bench scales: (1) one-step outflow test for primary drainage; (2) one-step inflow test for spontaneous (primary) imbibition; (3) multistep in/outflow tests for hysteresis scanning loops. One critical common among those setups is that the overflow tanks are all weighed by electrical scales. By pumping water from those overflow tanks to the constant head tanks, the water tables in those tanks can be kept at constant hydraulic heads in order to apply pressure boundary conditions on those soil columns. The one-step and multistep in/outflow tests can be carried out by vertically vibrating the constant head tanks, as illustrated in Figure 18c. As the water tables in constant head tanks are always conserved, the recharge or discharge in the overflow tanks is always equal to the soil moisture variation in soil columns. Thus, the accumulative in/outflow could be determined by weighing the overflow tanks with time.

Moreover, it is also essential to note the importance of preventing any potential bias caused by evaporation. For example, Figure 18d shows that all constant heads and overflow tanks sketched in Figure 18a,b should be tightly covered by cling wraps with small pinholes to deliver atmospheric pressure. This prevention should be performed if the primary drainage or imbibition tests are carried out for several days or months. However, if multistep in/outflow tests are conducted in a short period (e.g., several hours or a day), it is usually unnecessary to cover the vibrating head overflow (VHO) system (see Figure 18c,e,f) by cling wraps. Additionally, if the geotechnical laboratories are temperature- and humidity-controlled, those preventions are also unnecessary.

### 5.2. Simultaneous Data-Logging Configurations

Figure 18d shows four overflow tanks separately weighted by four electrical bench scales in front of the four constant head tanks. The cling wraps were utilized to cover any open area on the constant head and overflow tanks to prevent evaporations. In addition, small holes were pinned on cling wrap to maintain atmospheric pressure. The bench scales are Ohaus Ranger 3000 R31P30© with a 30 kg maximum capacity and a precision of ±1 kg. The water pump circulated water flow between constant head and overflow tanks. This water circulation process caused only a 1 kg variation within the scale precision.

All four scales were connected to a USB hub using four data transmission cables in the RS232-USB interface. The USB hub was then connected to a computer so the weight of overflow tanks could be consistently recorded using RsMulti^®^ Ver.1.10 W. The recording temporal resolution was set at 30 s using the driver and software supplied by Ohaus©. Since the valve between the soil column and the constant head tank was fully opened, the soil water could be drained out of a column to the constant head tank. As the constant head tank maintained the water head by discharging overflow, the overflow tank then collected all discharges from the constant head tank and simultaneously measured by each bench scale. In such a process, the measurement of the accumulative outflow from each soil column could be achieved.

Last but not least, even though there are no difficulties in applying those bench scales in measuring the accumulative overflow, there is still one tricky setting that needs to be carefully dealt with. As there is no data storage in each scale, all weighing data has to be immediately transferred into a computer. The frequency of sending weighing data from those bench scales to computers can be adjusted using a graphical interface in the Ohaus driver and software. To ensure your temporal resolution, such a data-sending frequency should be adjusted less or equal to your temporal resolution (e.g., 30 s in this work). If this frequency is adjusted to very high (e.g., multiple times per second) for only one scale, it should properly function. However, when numerous bench scales are connected to a computer, such high data-sending frequency will lead to the computer’s blue screen. Any operating system rebooting will not help resolve this issue unless each scale is independently connected for a frequency readjustment using the Ohaus graphic interface.

### 5.3. Post Data/Signal Processing

The RsMulti^®^ Ver. 1.10 W recorded the outputs from Ohaus electrical bench scales in an Excel spreadsheet. It is not difficult to straightforwardly plot the accumulative outflow by time. One tricky setting by using the RsMulti^®^ Ver. 1.10 W is that all weighing numbers have to come with the unit of mass in grams, resulting in strings instead of scale values in the output spreadsheet. Such a string spreadsheet cannot be directly analysed, so it requires a transformation between string and scale by eliminating the unit “g” in each cell using Matlab. After such a spreadsheet transformation, all data will be fully accessible for in/outflow analysis.

**Figure 19:**
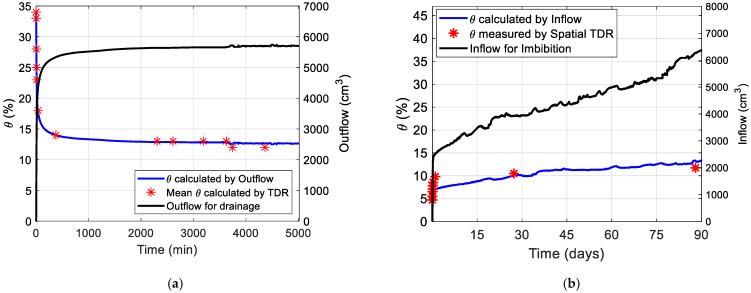
The validation between the average soil moisture contents measured by the conventional point-wise TDR technique and the average soil moisture contents calculated from accumulative outflow logging: (**a**) a demo of outflow; (**b**) a demo of inflow [49].

As aforementioned, the spatial TDR sensor-FRC can also be applied to the point-wise measurement of each column’s average soil moisture content using the travel times identified by the dual-tangent method, followed by capacitance and apparent permittivity calculations according to Figure 11a,b. With an aim to validate the accuracy of spatial TDR measurement, the accumulative outflow consistently recorded by the outflow logging system can be used to back-calculate the average soil moisture content of each soil column with the known soil packing conditions in Table 1. The accumulative in/outflow logging data sets for sand column tests are separately shown in Figure 19a,b, with the validations against the average soil moisture contents measured by the conventional point-wise TDR technique. In Figure 19, the discrete average soil moisture contents for each soil column measured by TDR almost overlap with the inversely calculated average soil moisture contents from the accumulative outflow logging in continuity. In summary, there is a good agreement between the accumulative in/outflow measured by bench scales and recharge/discharge of soil moisture content detected by the FRC sensor working on the point-wise scale. Both sensor techniques succeeded in mutual validations.

## 6. Conclusions

Although many unsaturated soil experiments have successfully delivered positive outcomes, most studies just concisely illustrated sensor techniques because their main objectives focused on bridging research gaps. Inexperienced research fellows might rarely follow up those techniques, so they could encounter very trivial and skill-demanding difficulties, undermining the quality of experimental outcomes. With an aim to salvage any fit into those cases, this work introduces technical challenges in applying three sensor techniques: high precision tensiometer, spatial time-domain reflectometry (Spatial TDR) and digital bench scales, which were used to measure three fundamental variables: soil suction, moisture content and accumulative outflow. The user experience encountered in applications is comprehensively elaborated from five perspectives: the functional mechanism, assembling/manufacturing approaches, installation procedure, simultaneous data-logging configurations and post data/signal processing. 

In conclusion, the technical challenges for applying those three sensor techniques are summarised in the following points:

(1) Soil suction measurement using high precision tensiometers:
  - The high precision tensiometers rely on measuring the voltage variations in the sensor body with the embedded Teflon membrane deforming under hydraulic pressures;
  - Manual refilling tensiometers is a prolonged process and also increases the risks of refilling failure, so a newly developed automatically refilling tensiometer is highly recommended for simultaneously refilling multiple tensiometers;
  - Tensiometers assembly could be better accomplished under the deaerated water than in the air with a few water drops on both shaft and sensor body;
  - There is no hydraulic offset between the sensor body and the ceramic tip on the acrylic shaft if it is horizontally installed, whereas the hydraulic offset should be adjusted back using trigonometric functions if it is not horizontally installed;
  - Commercial tensiometers can be connected to appending data loggers supplied by the supplier without any specific calibrations, and other data loggers having four-pin analogue channels can still be compatible with this sensor but demand specific water pressure-voltage signal calibrations;
  - Such a specific calibration could be achieved on both positive and negative pressure ranges using a U-shape manometer and the hanging column test, respectively;
  - With the Teflon membrane aging, there might be a substantial offset for older sensors lacking adequate maintenance, which also needs to be determined by the specific calibration mentioned above;
  - Theoretical soil suction heads, which are the vertical distance above the groundwater table, can be used to validate readings from all sensors installed on a soil column after primary drainage tests are finished.
(2) Soil moisture content profiles measurement using spatial TDR technique:
  - The spatial TDR technique upgrades the conventional TDR technique from a point-wise measurement to a profile detection by incorporating a flat ribbon cable (FRC) sensor with a fast TDR inversion analysis;
  - The self-made FRC sensors require prudently manufacturing skills to avoid any gas trapping, epoxy isolation leakage, etc., and the dimensions of the FRC sensor body and epoxy-packed terminals need to be accurately recorded as essential inputting parameters for the fast TDR inversion analysis;
  - The FRC sensor should be usually inserted into the centre of a soil column with a standard soil moisture-apparent permittivity calibration, but it can also be attached to the side of a soil column with a very specific calibration for such a case;
  - The spatial TDR data logging system consists of the TDR device, multiplexer and data logger, each of which can be accessed commercially, but specific data logging programs need to be coded using Basic language for applying self-made FRC sensors;
  - Due to a possible malfunctioning multiplexer, the TDR waveforms outputted from this data logging system might be undermined in terms of reflection coefficients but are still applicable for the conventional dual-tangent analysis, so a correction step is introduced in this work to rescue those TDR waveforms in terms of reflection coefficients for the fast TDR inversion analysis;
  - By achieving a good agreement between the simulated and measured TDR waveforms using the TDR inversion analysis, the spatial TDR technique can measure soil moisture profiles with a minimum temporal resolution of thirty seconds.
(3) Soil column accumulative in/outflow measurement using digital bench scales:
  - In comparison to electromagnetic flowmeter in high precision as well as high cost, the accumulative in/outflow given by weighing overflow tanks using electrical bench scales is a cost-effective solution, particularly for unsaturated soil water flow;
  - With different setups of constant and varying water heads at the soil column bottom, this measuring system can be applied to study one-step and multistep in/outflow on various hydraulic loading paths;
  - As the overflow tank weight can be sent from an electrical scale to a computer, the data-sending frequency should be adjusted to equal to or less than the logging temporal resolution. Otherwise, several digital scales simultaneously sending data to the computer in high frequencies will cause the computer operating system to fail in the form of a hardware blue screen;
  - According to validations between spatial TDR and digital scale measurements, this electrical bench scale logging system succeeded in measuring accumulative in/outflow for primary drainage and imbibition tests for several days or months.

Overall, this work aims to facilitate any new research fellows who carry out laboratory-scale soil column tests using the three sensors mentioned above. Additionally, it is anticipated that this work will help researchers bypass those issues to focus more on their research interests than the sensor technical details.

## 7. Patents

The patent for the T5 tensiometer is from UMS [65] GmbH D-81389 München Gmunderstr. 37, Germany. The patent for the spatial time domain reflectometry (spatial TDR) technique is from the Soil Moisture Group (SMG) at the University of Karlsruhe, Germany. The patent for TDR 100 (TDR signal generator and receiver), multiplexer (TDR multichannel connector) and CR1000 measurement and control system (TDR datalogger) are from Campbell Scientific, Inc. (CSI), USA. The patent for digital bench scales (Ohaus Ranger 3000 R31P30) is from Ohaus Ranger warehouse, Australia.

## Figures and Tables

**Table 1:** Grading analysis for GSD of selected medium sand and density control in soil columns.

Specifications	*d_50_* (mm)	*C_u_*	*C_c_*	*G_s_*	*ρ_dry,max_* (g/cm^3^)	*ρ_dry,min_* (g/cm^3^)	*ρ_dry_* (g/cm^3^)	*n* (%)	USCS
Values	0.42	1.50	1.00	2.65	1.80	1.50	1.75	34	SP

**Table 2:** The physical settings of FRC sensors in the TDR waveform logging program.

Wave Average (Times)	Propagation Velocity (Vp, the Ratio of C ^1^)	Data Points Number	Propagation Cable Length (m)	Propagation Measuring Window Length (m)	Probe Length (m)	Probe Offset (m)	Time Step (min)
10	0.67	1000	4.8	4.8	1.45	0	1

^1^ Noted that C is the speed of light/electromagnetic wave propagation.

**Table 3:** The parameters specification of the FRC sensor in the fast TDR inversion analysis.

Sensor Parameters	Values
Length of the sensor in polyethene and soil (*L_p_*)	1.40 m
Length of the sensor in epoxy (*L_a_*, *L_e_*)	0.025 m
The spatial resolution of inversion (Δx)	0.01 m
*C* of epoxy per unit length (*C_a_*, *C_e_*)	4 × 10^−11^ F/m
*C* of coaxial cable per unit length (*C_Z_*)	1 × 10^−10^ F/m
*L* of coaxial cable per unit length (*L_Z_*)	2.5 × 10^−7^ H/m
Sensor terminals configuration	Permanent cable connected
Inversion Type (two-way TDR tracing)	2*T_travel_* *, *C* and *G* reconstructions
*L* of sensor per unit length (*L = L_0_*)	7.56 × 10^−7^ H/m
*R* of sensor per unit length	0 Ohm/m
Capacitance model value *C_1_*	3.4 ×10^−12^ F/m
Capacitance model value *C_2_*	3.23 × 10^−10^ F/m
Capacitance model value *C_3_*	1.48 × 10^−11^ F/m
Lowest Capacitance *C_min_*	1.7552 × 10^−11^ F/m
Highest Capacitance *C_max_*	2.5717 × 10^−10^ F/m
Lowest Conductance *G_min_*	0 S/m
Highest Conductance *G_max_*	1 S/m
Initial Capacitance *C_initial_*	*C_initial_* *
Initial Conductance	0 S/m

* Noted that *T_travel_* is the travel time between two inflection points manually identified by the dual-tangent method to determine the average moisture content of a soil column; * the *C_initial_* is calculated according to Figure 11b.

## Data Availability

Available on request from the corresponding author.
